# Effect of Authigenic Chlorite on the Pore Structure of Tight Clastic Reservoir in Songliao Basin

**DOI:** 10.3390/ijerph20021406

**Published:** 2023-01-12

**Authors:** Yangchen Zhang, Xiyu Qu, Changsheng Miao, Jianfeng Zhu, Wen Xu, Weiming Wang

**Affiliations:** 1 School of Geosciences, China University of Petroleum, Qingdao 266580, China; 2 School of Prospecting and Surveying Engineering, Changchun Institute of Technology, Changchun 130021, China; 3 Sinopec Northeast Oil and Gas Branch, Changchun 130061, China

**Keywords:** authigenic chlorite, pore structure, petrophysical properties, tight sandstone, high pressure mercury injection, low temperature N_2_ adsorption

## Abstract

Authigenic chlorite is a common clay mineral in clastic rock reservoirs, and it has an important influence on the pore structure of tight clastic rock reservoirs. In this paper, the tight clastic reservoirs in the Lower Cretaceous Yingcheng Formation in the Longfengshan subsag in the Changling fault depression in the Songliao Basin were investigated. Polarized light microscopy, scanning electron microscopy (SEM), X-ray diffraction (XRD), high-pressure mercury injection (HPMI), and low temperature nitrogen adsorption (LTNA) were used to study the influence of authigenic chlorite on the pore structure of tight clastic reservoirs. The results show that the authigenic chlorite in the study area was mainly generated in the form of pore linings. The formation of the authigenic chlorite was mainly controlled by the parent rock type and the sedimentary microfacies in the provenance area. The hydrolysis and dissolution of the iron- and magnesium-rich intermediate-mafic magmatic rocks and the high-energy, open, weakly alkaline reducing environment in the delta-front underwater distributary channel were the key factors controlling the formation of the authigenic chlorite in the study area. The pore-lining chlorite slowed down compaction and inhibited quartz overgrowth, protecting the original pores. Moreover, there are a large number of intercrystalline pores in the chlorite, which provided channels for the flow of acidic water and thus the formation of secondary pores, playing a positive role in the physical properties of the tight clastic rock reservoirs. However, the pore-filling chlorite also blocked the pore throats, playing a negative role in the physical properties of the tight clastic rock reservoirs. The tight clastic rock reservoirs with pore-lining chlorite generally had low displacement pressures and large pore throat radii. The morphology of the nano-scale pores was mainly parallel plate-shaped slit pores. There were many primary pores and a small number of secondary pores in the reservoir. Some of the pores were connected by narrow-necked or curved sheet-like throats, and the pore structure was relatively good. A higher relative content of chlorite led to a larger nano-scale pore throat radius, a smaller specific surface area, a smoother pore surface, and stronger homogeneity. Authigenic chlorite played a positive role in the formation of the tight clastic reservoirs in the study area.

## 1. Introduction

Authigenic chlorite is a common diagenetic mineral in clastic reservoirs, and its content, production status, and spatial distribution are of great significance to the preservation of primary pores in reservoirs [1,2]. The influence of authigenic chlorite on reservoirs has always been a hot research topic. As early as the 1950s, when Heald was studying authigenic minerals in West Virginia sandstone, he noticed that authigenic chlorite occurred around detrital grains or around early quartz growths, and he concluded that the chlorite originated from the initial sediments [3]. At present, the research conducted on the influence of authigenic chlorite on reservoirs is mainly focused on conventional reservoirs, and researchers have different views on the influences of authigenic chlorite on reservoirs. Most researchers believe that chlorite coatings and a certain thickness of pore-lining chlorite play a constructive role in the preservation and transformation of primary pores [4,5], and chlorite can increase a reservoir’s resistance to compaction [6,7,8,9,10,11]. This is beneficial to the inhibition of quartz overgrowths [6,7,8,12] and carbonate cementation [9,10] and can protect the pores of the reservoir. However, some researchers believe that chlorite surrounding clastic grains reduces the original porosity, blocks the pore throats, and has a negative impact on the physical properties of a reservoir [13,14,15].

With the progress of oil and gas exploration and development and the continuous improvement of technology, the proportion of tight oil and gas produced has continued to increase, and the formation mechanism of tight clastic reservoirs has become a hot research topic. Previous studies have suggested that the existence of an abnormally high-porosity zone in middle-deep clastic reservoirs is related to the development of grain-coating chlorite [16,17,18,19]. Sun et al. [20] studied the relationship between authigenic chlorite and high-quality tight sandstone reservoirs and determined that chlorite has a good protective effect on primary pores and that a continuous reservoir with sufficient chlorite rim thickness can become a high-quality reservoir. Wu et al. [21] studied the influence of authigenic chlorite on the physical properties of tight oil reservoirs and proposed that chlorite linings can improve the compressive strength of sandstone and are beneficial to the preservation of pores and pore throats. In addition, they reported that chlorite linings more than 7 μm thick can effectively inhibit quartz overgrowth, which is favorable to the preservation of primary pores, and this allows the pore water to effectively make contact with the surrounding rock grains, which is beneficial to late-stage dissolution. At present, most researchers believe that pore-lining chlorite preserves primary pores by slowing down compaction and inhibiting quartz overgrowth, thereby improving the quality of tight clastic rock reservoirs and playing a constructive role in the formation of tight clastic rock reservoirs. However, pore-filling chlorite blocks the pore throats, thereby worsening the physical properties of tight clastic rock reservoirs [22]. Previous research on chlorite was limited to the study of the influence of chlorite cement on macroscopic physical properties, neglecting the study of the influence of chlorite on the microscopic pore throat structure of a reservoir. For tight clastic reservoirs, micro- and nano-scale pore throats are a very important part of the pores. The micro-pore structure is one of the key factors that controls the final development results of low-permeability and ultra-low-permeability sandstone reservoirs. The influence of authigenic chlorite on the microscopic pore structure of tight clastic rock reservoirs is not yet clear, and further research is urgently needed.

In this study, the tight clastic reservoirs in the Lower Cretaceous Yingcheng Formation in the Longfengshan subsag in the Changling fault depression in the Songliao Basin were investigated. Polarized light microscopy, scanning electron microscopy (SEM), quantitative X-ray diffraction (XRD), high-pressure mercury injection (HPMI), and low-temperature nitrogen adsorption (LTNA) were used to study the occurrence and origin of the authigenic chlorite in the reservoir rocks and the influence of the authigenic chlorite on the physical properties of the tight clastic reservoirs, as well as to innovatively investigate the effect of authigenic chlorite on the pore structure of the tight clastic reservoirs in order to provide a theoretical basis for the prediction of favorable tight clastic rock reservoirs and to provide a basis for further exploration and development.

## 2. Geological Setting

**Figure 1:**
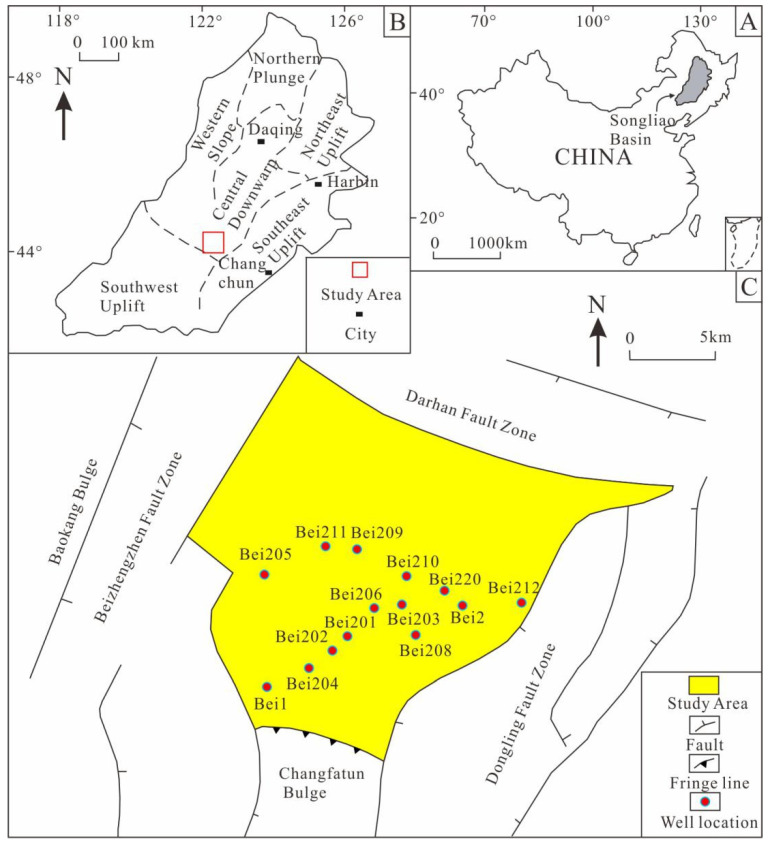
Geographical location of the Longfengshan subsag. (**A**) Location of the Songliao Basin. (**B**) Location of the Longfengshan subsag in the Songliao Basin. (**C**) Well location distribution in the Longfengshan subsag.

The Longfengshan subsag is located in the southern part of the central depression area of the Songliao Basin. It is adjacent to the Darhan Fault Zone in the north, the Changfatun bulge in the south, the Beizhengzhen Fault Zone in the west, and the Dongling Fault Zone in the east, and it has an area of nearly 300 km^2^ [23] (Figure 1). Due to the extensional strike-slip action of the Beizhengzhen Fault Zone and the Dongling Fault Zone, the fault activity in this area is strong.

**Figure 2:**
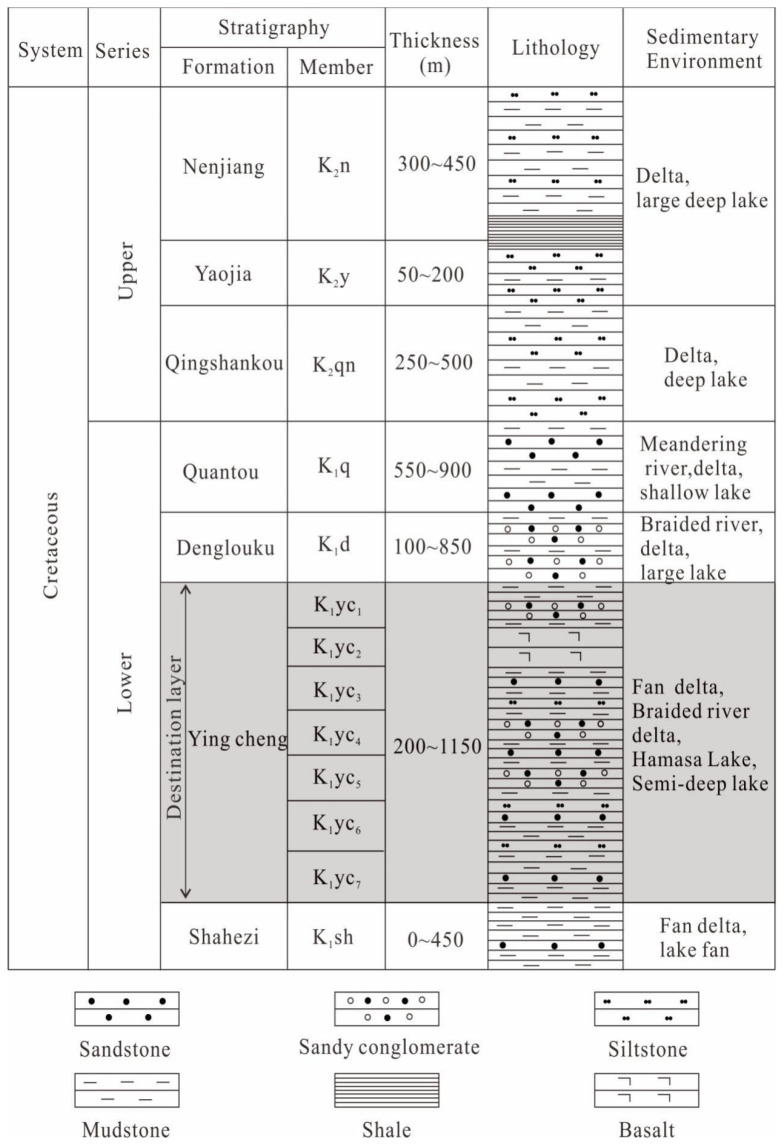
The Cretaceous stratigraphic sequence of the Longfengshan subsag.

The Lower Cretaceous strata in the Longfengshan subsag include the Shahezi, Yingcheng, Denglouku, and Quantou formations. The lithology is mainly fine sandstone, mudstone, and glutenite; basalt is present in the second member of the Yingcheng Formation. From bottom to top, the Upper Cretaceous strata include the Qingshankou, Yaojia, and Nenjiang formations. The lithology is mainly siltstone and mudstone, and a small section of dark shale is present in the Nenjiang Formation (Figure 2).

The target layer investigated in this study is the Lower Cretaceous Yingcheng Formation, which contains fan deltas, braided river deltas, shallow lakes, and semi-deep lake deposits. Overall, the study area was a delta sedimentary environment with strong hydrodynamics. The grain size distribution is wide, with medium-fine conglomerate, glutenite, medium-coarse sandstone, fine-siltstone, and mudstone developed [24]. The strata are mainly interbedded sandstone, conglomerate, and mudstone with different thicknesses. The bottom of the Yingcheng Formation is in pseudo conformable contact with the Shahezi Formation. The top of the Yingcheng Formation has been subjected to various degrees of weathering and denudation, and it is in pseudo conformable contact with the bottom of the Denglouku Formation [25]. Overall, the reservoirs in the Yingcheng Formation are tight, and gas production is dominant.

## 3. Materials and Methods

In total, 131 standard core plug samples (2.5 cm in diameter) of tight clastic rock were collected from the Longfengshan subsag in the Changling fault depression in the Songliao Basin. The samples were from Yingcheng Formation cores from 10 wells (wells Bei 2, Bei 201, Bei 202, Bei 203, Bei 204, Bei 206, Bei 208, Bei 209, Bei 210, and Bei 211), and the sampling depth was 2368.28–4130.35 m. Before the analyses, ethanol and benzene were used to remove the oil in the core samples. The liquid-filled porosities of the 131 plug samples were measured using a KX-90G tight rock vacuum saturation device and an electronic balance, and the air permeabilities of the samples were measured using an ECK-III core permeability tester. The National Standard of the People’s Republic of China (GB/T 29172-2012) was used as the reference for the analytical procedures.

### 3.1. Optical Observations

One hundred and thirty-one blue epoxy-impregnated thin sections were prepared to observe the occurrence of authigenic chlorite, the sequence of the other diagenetic phenomena, and the characteristics of the pores. The entire impregnation process was carried out under a vacuum in order to remove any gas in the samples. The samples were dried at 50 °C, and the blue epoxy resin was cured at 60 °C and injected into the sample at a pressure of 50 MPa. After being polished to a thickness of 30 mm, the thin sections were dyed with alizarin red to identify the carbonate minerals [26]. A Zeiss (Axioplan 2 imaging, Oberkochen, Germany) microscope was used to carry out the optical observations of the thin sections. The optical observations of 10 typical samples were conducted using an S-4800 cold field-emission scanning electron microscope. The samples were glued to the sample holder and then placed in a drying oven at 50 °C for 24 h. After the surface dust was blown off with a bulb blower, the sample was placed in the vacuum coater, and a coating of gold was applied in order to make the sample conductive. After the sample preparation was completed, the sample was placed in the sample chamber of the scanning electron microscope, the air was pumped out, and the sample was observed. The Petroleum and Natural Gas Industry Standard of the People’s Republic of China (SY-T 5162-1997) was used as the reference for the analytical procedures.

### 3.2. X-ray Diffraction (XRD)

In this study, the relative clay mineral contents of a total of 35 samples were analyzed using XRD. An X’Pert PRO MPD X-ray diffractometer (PANalytical, Holland, Almelo, Holland) was used for the XRD analyses. The voltage of the X-ray tube was 40 KV, the current was 40 mA, and the scan step size was 0.02°. The samples were processed before performing the XRD analyses. Before the XRD analysis of the clay mineral content, the samples needed to be crushed to a grain size of less than 5 mm and soaked in deionized water. Then, the impurities were removed with hydrogen peroxide or by washing the sample repeatedly with deionized water. After the clay was suspended, the suspension with a grain size of less than 2 μm was collected and centrifuged to settle the particles. Then, the centrifuged samples were dried in a drying oven at 40 °C. The dried samples were ground with an agate mortar until no grains could be felt by hand. The National Standard of the People’s Republic of China (SY-T 5163-2010) was used as the reference for the analytical procedures.

### 3.3. High-Pressure Mercury Intrusion (HPMI) Analysis

HPMI analysis was conducted on a total of 14 samples for this study. An AutoPore IV 9505 porosimeter (Micromeritics, USA, Shanghai, China) was used for the HPMI analyses, and the measurable pore-size distribution range was 0.003–630 µm. Before the HPMI analyses, the oil in the samples was removed, and 2–3 g of 2–3 mm grains was selected and dried at a temperature of 110 °C. Then, the sample was loaded into a dilatometer with a volume of 1 cm^3^. This process was carried out in a glove box filled with N_2_. Finally, the dilatometer containing the sample was transferred to the porosimeter for vacuum degassing under low pressure, the liquid mercury was injected, and the pore detection was performed under high pressure. The National Standard of the People’s Republic of China (GB/T 29171-2012) was used as the reference for the analytical procedures.

### 3.4. Low Temperature N_2_ Adsorption (LTNA) Analysis

The LTNA analysis was carried out on a total of 14 samples for this study. An Autosorb-iQ specific surface area and pore-size distribution analyzer (Quantachrome, USA, Atlanta, GA, USA) was used for the LTNA analyses. The pore-size distribution range of this instrument is 1.7–300 nm, and a specific surface area as low as 0.0005 m^2^/g can be measured. In order to eliminate the residual bound water and capillary water in the samples, before the LTNA analyses, all of the samples were pretreated in a high-temperature vacuum at 300 °C for 3 h. Then, high-purity N_2_ (>99.999%) was used as the adsorbate, and the N_2_ adsorption capacities of the samples under different relative pressures were measured at a temperature of 77 K. The National Standard of the People’s Republic of China (GB/T19587-2004) was used as the reference for the analytical procedures [27].

## 4. Results

### 4.1. Occurrence and Formation Period of Authigenic Chlorite

**Figure 3:**
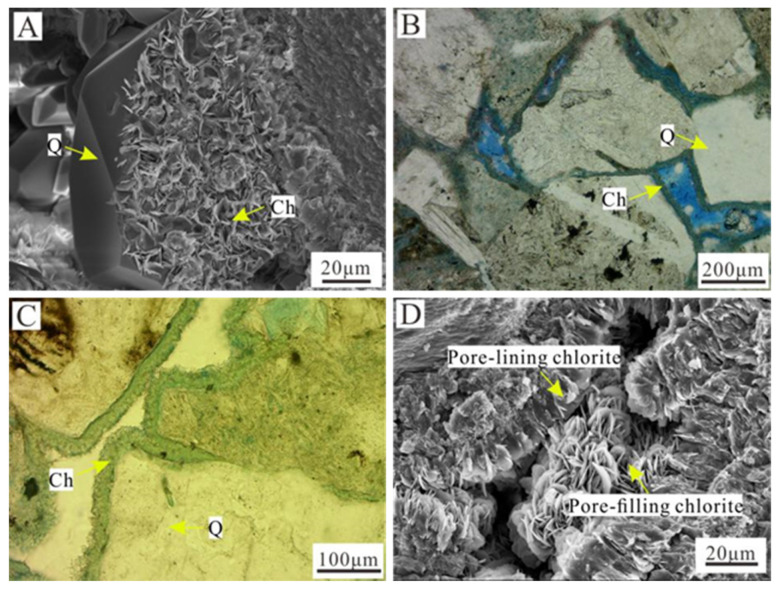
Occurrence of authigenic chlorite in Longfengshan subsag. (**A**) Chlorite coating, well Bei 202, 3087.48 m, SEM. (**B**) Pore-lining chlorite, well Bei 202, 3117.8 m, plane-polarized light. (**C**) Pore-lining chlorite, well Bei 210, 3949.75 m, plane-polarized light. (**D**) Pore-lining chlorite and pore-filling chlorite, well Bei 202, 3113.28 m, SEM. Ch—Chlorite; Q—Quartz.

The authigenic chlorite in the study area had three occurrence states. (1) Grain coatings (Figure 3A) grew on the surfaces of vertical grains, and they were usually less than 1 μm thick, covering the surfaces of the grains. The contact sites of the grains were distributed parallel to the grains due to extrusion. The chlorite crystals were needle-like, flakey or honeycombed [5]. (2) Pore-lining chlorite was the main form of authigenic chlorite in the study area (Figure 3B–D). Generally, it was developed at the pore edges outside of the grain contact point and grew in a ctenoid shape. The chlorite crystals were needle-like or bamboo leaf-shaped [7,21,28]. The thickness of these pore linings was 5–15 μm, and the pore-lining chlorite in well Bei 202 was the most developed and had the largest thickness (average of 15 μm) and strongest continuity. (3) Pore-filling chlorite: the intergranular pores were filled with bobble-shaped, rose-shaped, or dispersed monomers, and the crystal growth direction was non-directional [21] (Figure 3D).

**Figure 4:**
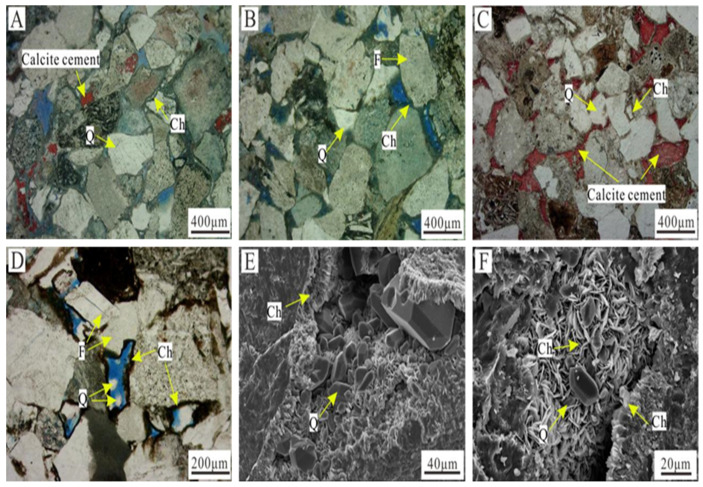
Relationship between authigenic chlorite and other diagenetic phenomena in Longfengshan subsag. (**A**) Pore-lining chlorite is developed, weak carbonate cementation, well Bei 202, 2994 m, plane-polarized light. (**B**) Pore-lining chlorite is developed, no carbonate cementation, well Bei 202, 3098.9 m, plane-polarized light. (**C**) Pore-lining chlorite and carbonate cementation develops, carbonate cementation is later than pore-lining chlorite, well Bei 206, 3363.77 m, plane-polarized light. (**D**) The intergranular pores surrounded by pore-lining chlorite are filled with authigenic quartz, well Bei 201, 3398.01 m, plane-polarized light. (**E**) The intergranular pores surrounded by pore-lining chlorite are filled with authigenic quartz, well Bei 202, 3075.43 m, SEM. (**F**) Pore-filling chlorite coating part of authigenic quartz, well Bei 202, 3113.53 m, SEM. Ch—Chlorite; Q—Quartz; F—Feldspar.

Based on the occurrence relationships between the different types of chlorite and the other diagenetic phenomena observed under the polarized light microscope, the formation periods of the different types of chlorite were determined. (1) The grain-coating chlorite films were relatively thin, were developed in the no-grain-contact stage before large-scale compaction, and were a product of the same sedimentary period. (2) In sandstone with pore-lining chlorite, the contact strength between the grains was weak, and point-to-line contact was generally dominant. In general, carbonate cementation was rarely developed (Figure 4A,B). Moreover, in the thin sections containing both pore-lining chlorite and carbonate cement, the carbonate cementation occurred after the pore-lining chlorite formed (Figure 4C). In addition, it was observed under the microscope that authigenic quartz (Figure 4D–F) was developed in the intergranular pores partially surrounded by the pore-lining chlorite (Figure 4D–F), indicating that the authigenic quartz filling occurred after the pore-lining chlorite formed. The above findings demonstrate that the pore-lining chlorite was formed in the early stage and was a product of the early diagenetic stage. (3) The pore-filling chlorite generally filled the intergranular pores in a rose-like shape, and the surface of the microcrystalline quartz within the intergranular pores was covered by a small amount of pore-filling chlorite (Figure 4F), indicating that the pore-filling chlorite formed after the authigenic quartz. Therefore, the pore-filling chlorite was formed in the last stage, and it was a product of the middle diagenetic stage.

### 4.2. Clay Mineralogy and Rock Type

The XRD analysis results of the relative clay mineral contents of the reservoir rocks in the study area are presented in Table 1. The types of clay minerals in the study area mainly included chlorite, illite, and an illite/montmorillonite mixed layer, while kaolinite was not observed. The contents of the three main clay minerals were quite similar. The relative content of chlorite ranged from 1% to 73%, with an average of 35.8%; the relative content of illite ranged from 13% to 66%, with an average of 32%; and the relative mineral content of the illite/montmorillonite mixed layer ranged from 10% to 64%, with an average of 32.2% (Table 1).

**Figure 5:**
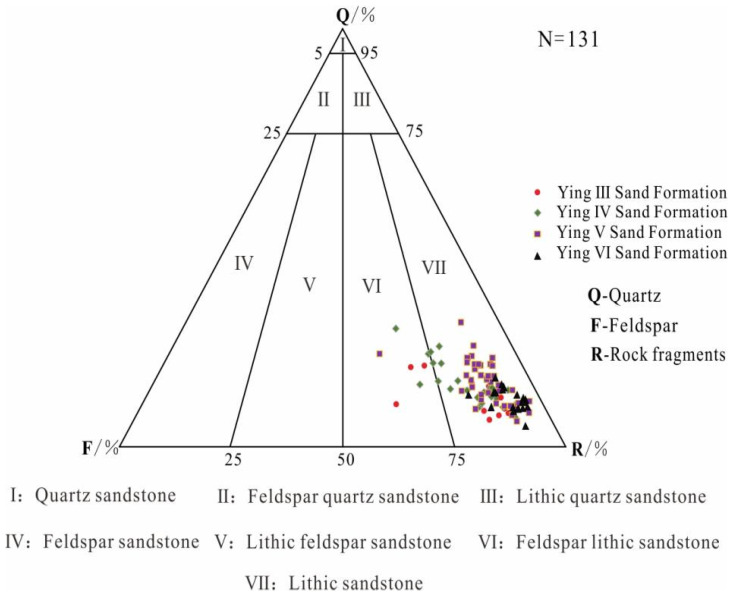
Lithological triangulation of tight clastic reservoirs in the study area.

According to the identification results of 131 cast thin sections, the skeletal particle types of the tight sandstone reservoir in the study area include quartz, feldspar, and rock fragments, with average contents of 14.2%, 14.9%, and 69.5%, respectively, and the rock type is mainly lithic sandstone, followed by feldspar lithic sandstone (Figure 5).

### 4.3. High-Pressure Mercury Injection Analysis

The results of the HPMI analysis revealed that the displacement pressure (P_d_) of the 14 samples from the study area ranged from 0.469 MPa to 5.51 MPa, with an average of 2.335 MPa. The median saturation pressure (P_50_) ranged from 11.149 MPa to 113.454 MPa, with an average of 47.655 MPa. The maximum pore throat radius (r_max_) ranged from 0.134 μm to 1.567 μm, with an average of 0.612 μm. The average pore throat radius (r^) ranged from 0.024 μm to 0.276 μm, with an average of 0.106 μm. The median radius of the pore throats (r_50_) ranged from 0.007 μm to 0.068 μm, with an average of 0.027 μm. The maximum mercury saturation (S_m_) ranged from 70.264% to 96.38%, with an average of 90.533%. The skewness ranged from −0.715 to 0.246, with an average of −0.270 (Table 2).

**Figure 6:**
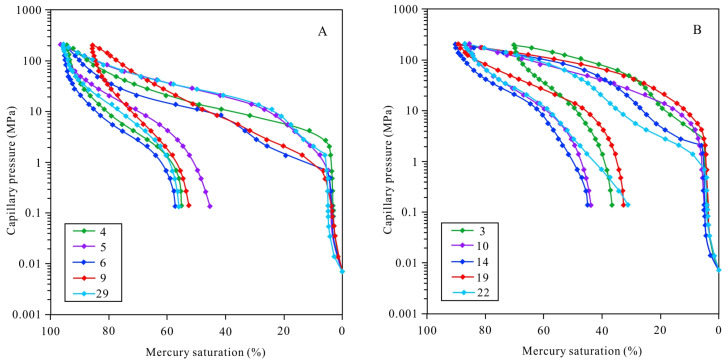
(**A**) HPMI curve of samples with Pore-lining chlorite. (**B**) HPMI curve of samples without Pore-lining chlorite.

**Figure 7:**
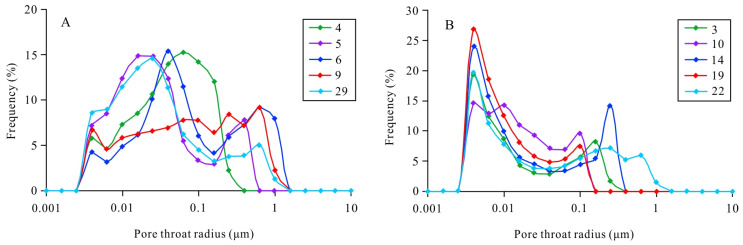
(**A**) Pore throat radius of samples with pore-lining chlorite. (**B**) Pore throat radius of samples without pore-lining chlorite.

The mercury intrusion and extrusion curves for several typical samples obtained from the HPMI analysis are shown in Figure 6. The displacement pressures (P_d_) of the samples containing pore-lining chlorite were relatively low, ranging from 0.469 MPa to 2.740 MPa. Their mercury intrusion saturations (S_m_) were relatively high, ranging from 85.454% to 95.99%. Their median saturation pressures (P_50_) were relatively low, ranging from 11.149 MPa to 27.681 MPa. The samples without pore-lining chlorite had relatively high pressures (P_d_), ranging from 0.674 MPa to 5.51 MPa. Their mercury intrusion saturations (S_m_) were relatively low, ranging from 70.264% to 96.38%. The median saturation pressures (P_50_) were relatively high, ranging from 35.827 MPa to 113.454 MPa (Figure 6) (Table 2). The pore throat radius distributions of the samples containing pore-lining chlorite were uniform, and the peak value of the pore throat radii was greater than 0.01 μm (Figure 7A). The pore throat radius distributions of the samples without pore-lining chlorite were relatively concentrated, exhibiting a bimodal shape, and the pore throat radius was small, mainly concentrated below 0.01 μm (Figure 7B).

### 4.4. Low-Temperature N_2_ Adsorption Analysis

The results of the LTNA revealed that the total pore volumes of the 14 samples from the study area ranged from 0.009 mL/g to 0.0196 mL/g, with an average of 0.0133 mL/g. Their BET (Brunauer, Emmett, and Teller) specific surface areas ranged from 1.5252 m^2^/g to 9.3627 m^2^/g, with an average of 4.4328 m^2^/g. The average diameter of their BJH (Barrett–Joyner–Halenda) adsorptions ranged from 10.9172 nm to 17.1693 nm, with an average of 13.2533 nm. Their fractal dimensions ranged from 2.3921 to 2.6975, with an average of 2.5526 (Table 3).

**Figure 8:**
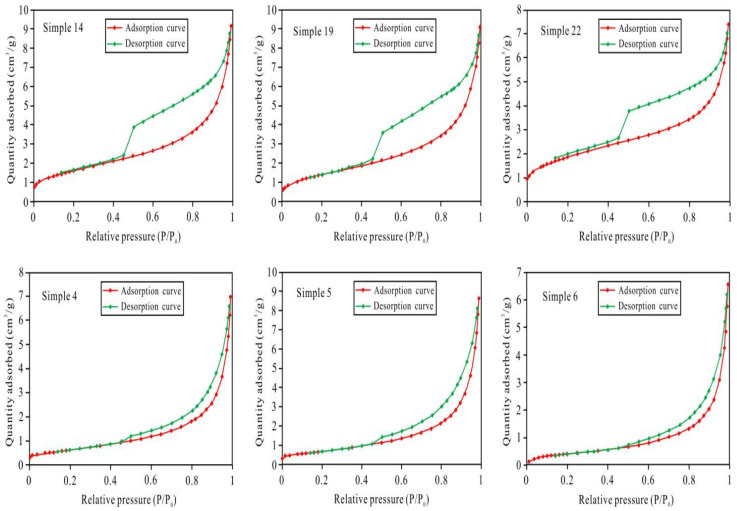
Adsorption–desorption curves of a part of the sample.

When the relative pressure was high (P/P_0_ > 0.4), the sample adsorption and desorption curves no longer overlapped, resulting in a hysteresis loop. According to the IUPAC (International Union of Pure and Applied Chemistry) classification standard, the low-temperature N_2_ adsorption–desorption curves of the samples from the study area are basically H2- and H3-type curves (Figure 8), and the corresponding pore throat shapes are ink-bottle-shaped pores and slit-shaped pores, respectively. The samples with relatively well-developed pore-lining chlorite (e.g., samples 4, 5, and 6) generally had good physical properties. Their low-temperature nitrogen adsorption–desorption curves were H3-type curves, and the pore throat shape was parallel plate slit-type. Thus, the nano-scale pore throats were the main throats, and the parallel plate-shaped slit throats connected the relatively large pores. In the samples without pore-lining chlorite (e.g., samples 14, 19, and 22), the intergranular pores were generally filled with other types of cements, and the physical properties were generally poor. The low-temperature nitrogen adsorption–desorption curves were H2-type curves, and the pore throats were ink bottle-shaped pores with narrow mouths and wide bodies. The wide body parts were nano-pores, and the narrow mouths were the throats.

## 5. Discussion

### 5.1. Formation Mechanism of Authigenic Chlorite

**Figure 9:**
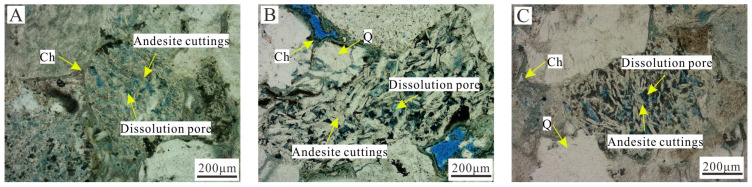
Pore-lining chlorite and andesite rock fragments internally dissolved pores. (**A**) Well Bei 210, 3949.97 m, plane-polarized light. (**B**) Well Bei 202, 3112.75 m, plane-polarized light. (**C**) Well Bei 210, 3949.5 m, plane-polarized light. Ch—Chlorite; Q—Quartz.

**Figure 10:**
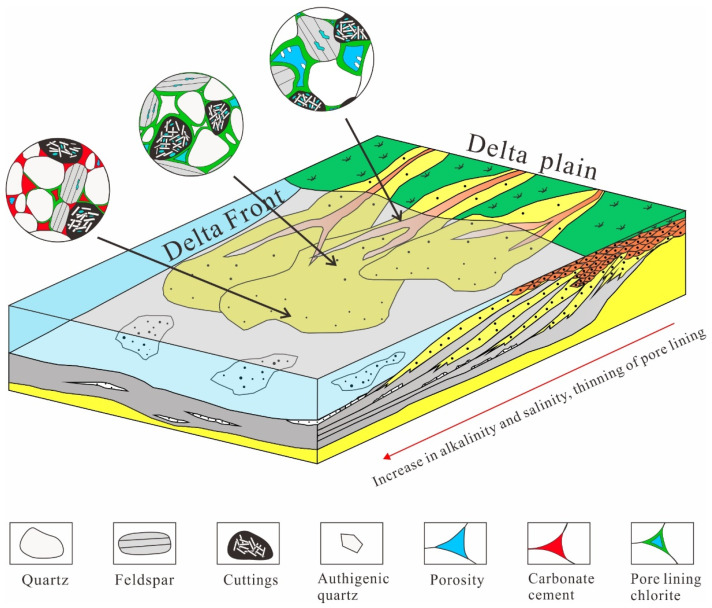
Relationship between authigenic chlorite and sedimentary environment (modified from [24]). The entrance of the lake is a high-energy, open, weakly alkaline reducing environment. The iron in the river flocculates due to the difference in salinity at the entrance of the lake. Under strong hydrodynamic conditions, clay minerals adhere to the surfaces of the particles to form pore-lining chlorite. As the water depth increases, the alkalinity and reducibility increase, the hydrodynamic conditions weaken, the pore-lining chlorite becomes thinner, and the carbonate cement precipitates from the pore fluid.

The formation of authigenic chlorite is affected by many factors, and a weakly alkaline (pH of 7–9) reducing environment rich in iron and magnesium is the key to the formation of authigenic chlorite [1,2,10]. The types of authigenic chlorites in the study area mainly had the following two formation mechanisms. (1) The influence of the parent rock type in the provenance area: Intermediate-mafic magmatic rock and metamorphic rock [23] were the main rock types in the provenance area during the deposition of the Yingcheng Formation in the Longfengshan subsag, and the main rock type deposited was lithic sandstone. Dark minerals, such as biotite and amphibole, and the iron ions produced during the hydrolysis and dissolution of a large amount of volcanic rock fragments provided the material basis for the formation of authigenic chlorite (Figure 9). (2) The controlling influence of the sedimentary microfacies: The study area mainly contained a fan delta sedimentary system, and the pore-lining chlorite was mainly developed in the underwater distributary channel microfacies of the fan delta front. Due to the strong hydrodynamic conditions of the underwater distributary channel microfacies, pore fluid migration occurred. As Bernoulli’s theory states, under the pressure generated by the local flow velocity over the grain surfaces, the clay was adsorbed onto the grain surfaces, forming pore-lining chlorite [22]. As the water body deepened, the alkalinity and reducibility increased, the hydrodynamic conditions weakened, and the pore fluid migration slowed. The development of the pore-lining chlorite became poor and basically ceased, and siliceous cement and late-stage ferrous carbonate cement easily precipitated from the pore fluid, thereby filling the intergranular pores and worsening the physical properties of the reservoir rocks (Figure 10).

### 5.2. Impact on Reservoir Physical Properties

**Figure 11:**
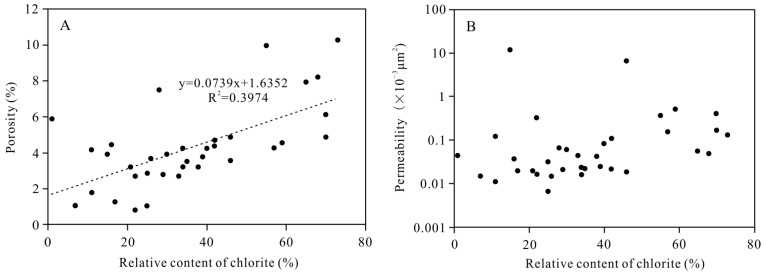
Relationship between relative content of chlorite and (**A**) porosity and (**B**) permeability.

The relative chlorite content is positively correlated with porosity (Figure 11A), but it is not significantly correlated with permeability (Figure 11B). Except for a few points, the higher the relative chlorite content, the greater the porosity. The observations under the polarized light microscope showed that there were generally a large number of primary pores in the thin sections containing well-developed pore-lining chlorite (Figure 4A,B), indicating that the increase in porosity was mainly caused by larger number of primary pores. The contribution of the authigenic chlorite to the physical properties of the tight clastic rock reservoirs is mainly reflected in the preservation of the primary pores by the pore-lining chlorite.

**Figure 12:**
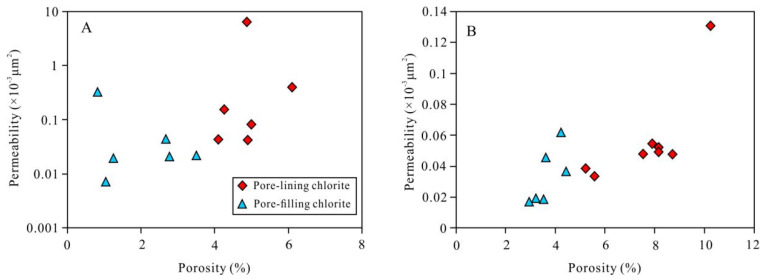
Relationship between porosity and permeability of chlorite in different occurrences: (**A**) well Bei 210 and (**B**) well Bei 202.

From a single-well point of view, in the reservoir rocks in which pore-lining chlorite and pore-filling chlorite developed at the same time, the sandstone containing pore-lining chlorite has a relatively high porosity. For example, the porosity of the sandstone containing pore-lining chlorite in well Bei 210 is greater than 4% (Figure 12A). Similarly, the porosity of the sandstone containing pore-lining chlorite in well Bei 202 is greater than 5% (Figure 12B). For the sandstone containing pore-filling chlorite, the porosity is low. For example, the porosities of the sandstones containing pore-filling chlorite in wells Bei 210 and Bei 202 are less than 4% and 5%, respectively (Figure 12A,B). The differences in the permeabilities of sandstones containing different types of chlorite are not as significant as in the differences in their porosities. The positive effect of pore-lining chlorite on the physical properties of tight clastic rocks is mainly reflected in its contribution to the porosity. The pore-filling chlorite had a negative effect on the physical properties of the tight clastic rock reservoirs, blocking the pores and throats and reducing the porosity and permeability.

### 5.3. The Influence of Authigenic Chlorite on Micro-Pore Structure

#### 5.3.1. Impact on Visual Pore Structure

**Figure 13:**
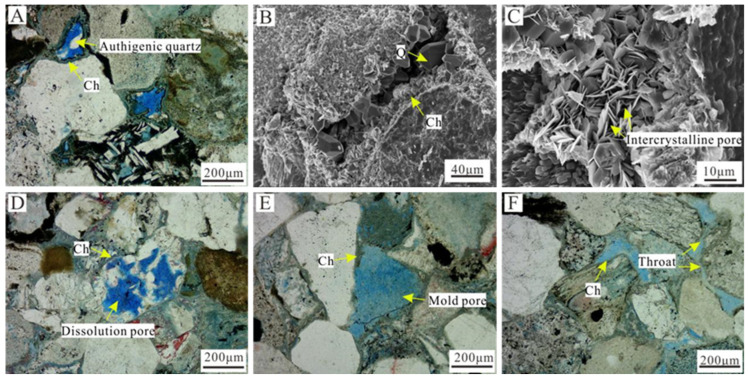
Relationship between authigenic chlorite and macroscopic pores. (**A**) Intergranular pores surrounded by pore-lining chlorite are filled with authigenic quartz, well Bei 202, 3112.75 m. (**B**) Intergranular pores surrounded by pore-lining chlorite are filled with authigenic quartz, well Bei 202, 3101.4 m. (**C**) Authigenic chlorite has a large number of intercrystalline pores, well Bei 202, 3125 m. (**D**) Intragranular dissolution pores of feldspar particles, well Bei 202, 3087.33 m. (**E**) Mold pore surrounded by pore-lining chlorite, well Bei 202, 2994 m. (**F**) The compaction effect is weak, and part of the throat is preserved, well Bei 202, 2994 m.

**Figure 14:**
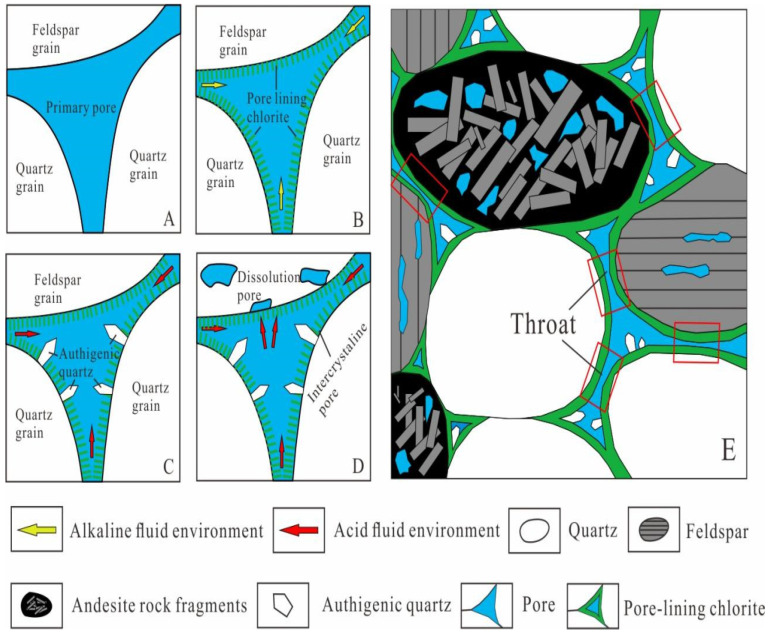
Influence of pore-lining chlorite on pore structure of tight clastic rock reservoir. (**A**) In the initial deposition stage, primary intergranular pores developed. (**B**) In the early diagenesis period, under weakly alkaline conditions, the pore-lining chlorite developed. (**C**) During the mid-diagenesis period, authigenic microcrystalline quartz gradually formed under acidic conditions. (**D**) The acidic fluid contacts the feldspar particles through the chlorite intercrystalline pores and then forms intragranular dissolved pores. (**E**) When the pore-lining chlorite is developed, the pore structure appears as large pores and narrow throats.

Before the beginning of diagenesis, the intergranular pores were mainly primary pores. As diagenesis progressed, when the fluid environment was an open, weakly alkaline reducing environment, a large amount of pore-lining chlorite developed with a good continuity, and the primary pores were surrounded by the pore-lining chlorite (Figure 13A and Figure 14B). In middle diagenetic stage A, the organic acids introduced by the oil and gas filling gradually changed the fluid environment from weakly alkaline to weakly acidic, and authigenic quartz began to form. The presence of the pore-lining chlorite inhibited the pressure dissolution between the quartz grains and reduced the SiO_2_ content of the pore fluid [20]. In addition, because the pore-lining chlorite surrounded the quartz grains and occupied the crystalline quartz basement [8], quartz overgrowth was suppressed. The quartz overgrowth phenomenon was basically not observed under the microscope. The quartz usually existed as authigenic crystals within the intergranular pores (Figure 13B and Figure 14C).

The pore-lining chlorite blocked part of the pore throats and wrapped around the grains, preventing the acidic fluid from coming into contact with the feldspar grains in a large area. However, due to the large number of nano-scale intercrystalline pores in the chlorite (Figure 13C), the throats were not completely closed, so the fluid could still come into contact with the particle surfaces through the intercrystalline pores. When the acidic fluid came into contact with the feldspar grains through the intergranular pores, the insides of the feldspar grains were dissolved, resulting in the formation of intragranular dissolution pores (Figure 13D and Figure 14D), which were further dissolved into mold holes surrounded by the pore-lining chlorite (Figure 13E).

The pore-lining chlorite was formed in the early stage, and it prevented the later compaction to a certain extent [20]. In the sandstones containing pore-lining chlorite, the grains were mainly in point–line contact, the particle contact strength was low, the primary pores were well preserved, and there were small quantities of narrow-necked and curved pore throats (Figure 13F). The pore-lining chlorite occupied a certain amount of the pore space, so the throats were narrow overall, and the pore throat structure was mainly characterized by large pores and fine throats (Figure 14E). Compared with other types of reservoirs with strong compaction or cementation, these reservoir rocks have a better pore structure.

#### 5.3.2. Impact on Micro-Pore Structure

**Figure 15:**
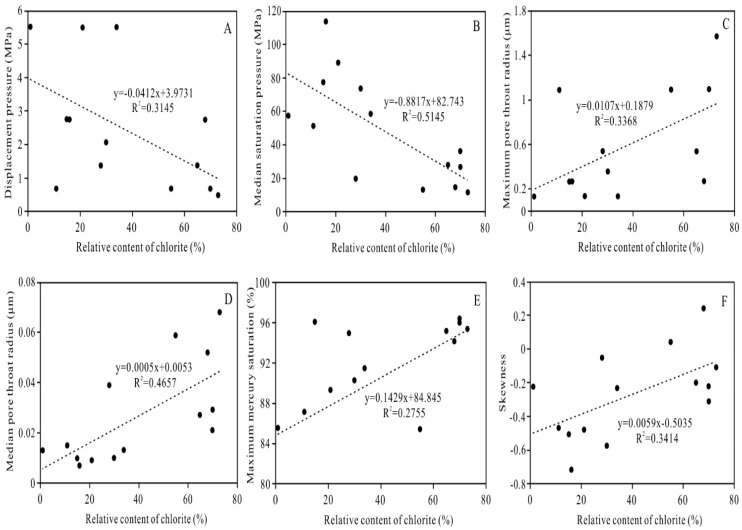
Relationship between the relative content of chlorite and (**A**) Displacement pressure, (**B**) Median saturation pressure, (**C**) Maximum pore throat radius, (**D**) Median pore throat radius, (**E**) Maximum mercury saturation, and (**F**) Skewness.

Based on the results of the HPMI analyses, the relative chlorite content is correlated with the displacement pressure, the median saturation pressure, the maximum pore throat radius, the median pore throat radius, the maximum mercury intrusion saturation, and the skewness. When the relative chlorite content is relatively high, the displacement pressure and the median saturation pressure are low (Figure 15A,B), the maximum pore throat radius and the median pore throat radius are relatively large (Figure 15C,D), the maximum intrusion mercury saturation is relatively high (Figure 15E), and the skewness is relatively large, with a slightly fine skewness and a slightly coarse skewness (Figure 15F). The above results reveal that under the background of overall tight reservoirs, clastic rocks with relatively well-developed pore-lining chlorite have a good connectivity and storage capacity, with a large micron-sized pore throat radius and a relatively good pore throat structure. These reservoirs have good storage properties.

#### 5.3.3. Impact on Nano-Scale Pore Structure

**Figure 16:**
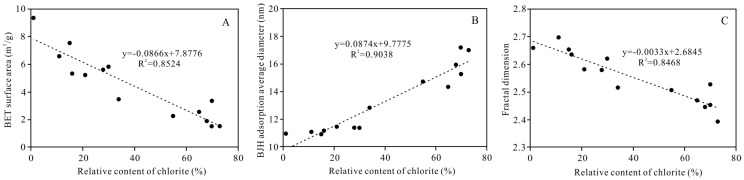
Relationship between the relative content of chlorite and (**A**) BET surface area, (**B**) BJH adsorption pore average diameter, and (**C**) fractal dimension.

Based on the results of the LTNA analyses and the BET specific surface area of the nano-scale pores of the tight clastic reservoirs in the study area, the average diameter obtained using the BJH adsorption method and the fractal dimension are strongly correlated with the relative chlorite content (Figure 16), indicating that the nano-scale pores in the reservoir rocks in the study area are mainly related to the intercrystalline pores in the clay minerals. As the relative chlorite content increases, the BET specific surface area gradually decreases, the average diameter obtained using the BJH adsorption method gradually increases, and the fractal dimension gradually decreases. A higher relative chlorite content in the clay minerals led to lower relative illite and illite/montmorillonite mixed layer contents. Because chlorite has a smaller specific surface area than illite and the illite/montmorillonite mixed layer [29], the specific surface area of the nano-scale pores composed of clay minerals was smaller, and thus, the methane gas adsorption capacity of the clay minerals in the reservoir was decreased, and methane gas was more likely to exist in the pores in a free state. As the relative chlorite content increased, the average pore diameter increased, indicating that when authigenic chlorite was present, the nano-scale pores were relatively large, and the proportions of mesopores and macropores in the authigenic chlorite were higher compared with those of non-authigenic chlorite. This is related to the large intercrystalline pores and is also another reason for the small specific surface area. The chlorite crystals were generally smooth and flaky, with regular intercrystalline pore shapes; the pore surfaces were smoother, and the pore structure was more uniform, so the fractal dimension was smaller.

## 6. Conclusions

The authigenic chlorite in the study area is mainly pore-lining chlorite, with a thickness of about 5–15 μm, which was formed in the early diagenetic stage.

The formation of authigenic chlorite was mainly controlled by the parent rock type and the sedimentary microfacies in the provenance area. The iron and magnesium ions produced by the hydrolysis and dissolution of iron- and magnesium-rich intermediate-mafic magmatic rocks were the material basis for the formation of the authigenic chlorite. The strong hydrodynamic conditions and open, weakly alkaline reducing environment were the key factors controlling the formation of the pore-lining chlorite.

The pore-lining chlorite played a constructive role in the physical properties of the tight clastic rock reservoirs. The pore-lining chlorite slowed down the compaction and inhibited the overgrowth of quartz. Additionally, because the pore-lining chlorite was formed in an open and high-energy environment, cements were not easily precipitated from the pore fluids, and intergranular pores were basically not filled by late-stage cements, so the primary pores were preserved. Furthermore, the chlorite contained a large number of intercrystalline pores, which provided channels for the flow of acidic water, thereby promoting dissolution and generating secondary pores.

The authigenic chlorite had a certain influence on the pore structure of the tight clastic rock reservoirs. The pore throat assemblage of reservoirs containing pore-lined chlorite is characterized by large pore spaces and fine throats. When pore-lined chlorite is developed, the pore throats are poorly sorted, with a wide distribution of pore throat radii and a high proportion of large pores. Nano-scale pore throats are usually parallel plate-like slit-like pores with a large pore throat radius, small specific surface area, smooth pore surface, and high uniformity.

## Figures and Tables

**Table 1:** Clay mineral composition base on XRD analysis.

Simple Number	Well	Depth (m)	Relative Content of Clay Minerals (%)			
			Chlorite	Illite	I/S Mixed Layer	I/S Mixed Layer Ratio
1	Bei2	3741.87	11	25	64	20
2	Bei201	3083.49	26	27	47	20
3	Bei202	3065.55	16	25	59	20
4	Bei202	3112.75	68	15	17	20
5	Bei202	3117.8	65	14	21	20
6	Bei202	3128.6	73	13	14	20
7	Bei202	3130.8	46	21	33	20
8	Bei203	3317	28	22	50	20
9	Bei203	3317.87	55	16	29	20
10	Bei204	2881.3	1	66	33	20
11	Bei206	3235.57	42	16	42	5
12	Bei206	3237.61	38	20	42	20
13	Bei206	3243.6	15	22	63	20
14	Bei206	3316.62	30	22	48	20
15	Bei206	3331.3	39	28	33	20
16	Bei206	3332.66	40	22	38	15
17	Bei206	3333.26	42	27	31	20
18	Bei206	3338	22	44	34	20
19	Bei206	3347.36	21	49	30	20
20	Bei206	3360	25	45	30	20
21	Bei206	3366	34	37	29	20
22	Bei208	3261.45	11	47	42	20
23	Bei208	3432.41	7	45	48	20
24	Bei209	3258.16	59	22	19	20
25	Bei209	3756.5	34	44	22	20
26	Bei210	3945.95	46	36	18	20
27	Bei210	3945.33	70	20	10	20
28	Bei210	3946.45	57	28	15	20
29	Bei210	3951.15	70	19	11	5
30	Bei210	4125.4	29	49	22	20
31	Bei210	4126.8	33	48	19	20
32	Bei210	4128.05	35	44	21	20
33	Bei210	4129.45	17	54	29	20
34	Bei210	4129.65	22	55	23	20
35	Bei210	4129.95	25	33	42	5

Note: I/S: Illite/Smectite.

**Table 2:** Parameters for microscopic pore structures drawn from the HPMI.

Sample Number	Well	Depth (m)	Occurrence	P_d_ (MPa)	P_50_ (MPa)	r_max_ (μm)	r^ (μm)	r_50_ (μm)	S_m_ (%)	Skewness
3	Bei202	3065.55	N/A	2.742	113.454	0.268	0.058	0.007	70.264	−0.715
4	Bei202	3112.75	Pore-lining	2.74	14.328	0.268	0.073	0.052	94.16	0.246
5	Bei202	3117.8	Pore-lining	1.365	27.681	0.539	0.101	0.027	95.14	−0.197
6	Bei202	3128.6	Pore-lining	0.469	11.149	1.567	0.276	0.068	95.354	−0.107
8	Bei203	3317	Pore-lining	1.364	19.602	0.539	0.128	0.039	94.929	−0.049
9	Bei203	3317.87	Pore-lining	0.674	12.865	1.09	0.207	0.059	85.454	0.046
10	Bei204	2881.3	N/A	5.51	57.051	0.133	0.028	0.013	85.54	−0.222
13	Bei206	3243.6	N/A	2.747	77.034	0.268	0.041	0.01	96.08	−0.506
14	Bei206	3316.62	N/A	2.056	73.362	0.357	0.096	0.01	90.268	−0.573
19	Bei206	3347.36	N/A	5.495	88.912	0.134	0.024	0.009	89.30	−0.476
21	Bei206	3366	N/A	5.5	58.244	0.134	0.03	0.013	91.454	−0.23
22	Bei208	3261.45	N/A	0.674	51.125	1.09	0.167	0.015	87.152	−0.467
27	Bei210	3945.33	N/A	0.674	35.827	1.09	0.123	0.021	96.38	−0.309
29	Bei210	3951.15	Pore-lining	0.673	26.541	1.093	0.132	0.029	95.99	−0.218

Note: P_d_: Displacement pressure; P_50_: Median saturation pressure; r_max_: Maximum pore throat radius; r^: Average pore throat radius; r_50_: Median pore throat radius; S_m_: Maximum mercury saturation; N/A: Undeveloped chlorite.

**Table 3:** Parameters for microscopic pore structures drawn from the LTNA.

Sample Number	Total Pore Volume (mL/g)	BET Specific Surface Area (m^2^/g)	BJH Adsorption Average Diameter (nm)	Fractal Dimension
3	0.0129	5.3246	11.1797	2.635
4	0.0109	1.91	15.9242	2.4455
5	0.0133	2.548	14.3299	2.4689
6	0.0101	1.5461	16.9608	2.3921
8	0.0158	5.6084	11.3891	2.5795
9	0.0107	2.2536	14.7151	2.5064
10	0.0196	9.3627	10.9425	2.6585
13	0.0156	7.5304	10.9172	2.6536
14	0.0141	5.8173	11.3749	2.6204
19	0.014	5.23	11.4678	2.5807
21	0.0128	3.4655	12.8308	2.5163
22	0.0114	6.5748	11.086	2.6975
27	0.0154	3.3625	15.2584	2.5276
29	0.009	1.5252	17.1693	2.4538

## Data Availability

All data are available from the corresponding author upon reasonable request.
